# Diazepam Reduces Escape and Increases Closed-Arms Exploration in Gerbils After 5 min in the Elevated Plus-Maze

**DOI:** 10.3389/fpsyg.2019.00748

**Published:** 2019-04-02

**Authors:** Javier Leonardo Rico, Luisa Fernanda Muñoz-Tabares, Marisol R. Lamprea, Camilo Hurtado-Parrado

**Affiliations:** ^1^Animal Behavior Laboratory, Fundación Universitaria Konrad Lorenz, Bogotá, Colombia; ^2^Neurosciences Laboratory, Psychology Department, Universidad Nacional de Colombia, Bogotá, Colombia; ^3^Department of Psychology, Troy University, Alabama, AL, United States

**Keywords:** elevated plus-maze, mongolian gerbil (*Meriones unguiculatus*), anxiety-related behavior, diazepam, factor analysis, ethopharmacology, escape

## Abstract

Despite the wide implementation of the elevated plus-maze (EPM) test to assess anxiety-related behaviors in rodents, the interpretation of these measures in gerbils has received limited attention. Here, male gerbils were treated with vehicle or diazepam, followed by a 20-min EPM session. EPM data were subjected to minute-by-minute, 5-min bins and factor analyses. During the first 5-min, gerbils avoided the closed arms in favor of the open arms and diazepam increased open-arms entries; furthermore, a single factor (escape behavior) explained all the analyzed measures. Only after 5-min, gerbils reduced open-arms exploration and three independent factors emerged for each subsequent 5-min bin. These findings suggest that EPM data from gerbils should be analyzed in at least two 5-min bins. Measures from the standard 5-min session seem to be related to an escape response from the EPM through the open arms. Once habituated, measures from the second 5-min bin seem to be related to a conflictive situation: keep trying to escape unsuccessfully (due to open-arms height) or seek protection in the closed arms (unsafe places). Diazepam seems to reduce this conflict by mitigating the escape response (Factor 1 – Anxiety) and increasing closed-arms approach (Factor 2) and risk assessment (Factor 3). Unlike mice and rats, a decrease in open-arms exploration and an increase in risk assessment could be interpreted as an anxiolytic-like effect in gerbils.

## Introduction

The elevated plus-maze (EPM) is a widely used test to evaluate anxiety-related behaviors and to detect anxiolytic properties of compounds in rodents (Carobrez and Bertoglio, 2005; Pawlak et al., 2012). Validated for rats and mice (Pellow et al., 1985; Lister, 1987), the test is based on the natural tendency of these rodents to avoid open spaces in favor of protected areas (Pellow et al., 1985; Lister, 1987; Rodgers et al., 1997; Pawlak et al., 2012). Rats and mice exposed to a 5-min session in the EPM spent more time in the closed arms of the apparatus, and the previous administration of anxiolytic compounds such as benzodiazepines increases open-arms exploration (for review see Carobrez and Bertoglio, 2005). Thus, the increase of entries and time spent in the open arms have been interpreted as a reduction of anxiety-related behaviors (Pellow et al., 1985; Lister, 1987; Rodgers et al., 1997; Pawlak et al., 2012).

The Mongolian gerbil has become a popular animal model used in research areas from gastric and neurological disease to animal cognition (for a review see Hurtado-Parrado et al., 2015). In contrast to rats and mice, gerbils are monogamous and diurnal/crepuscular species (Romero-Morales et al., 2018; Hurtado-Parrado et al., 2019). Furthermore, the fact that their NK1 receptors are closer in homology to the human NK1 receptor (Griffante et al., 2006; Leffler et al., 2009), makes this species a promising model for the study of anxiety disorder (Frick et al., 2015).

The validation of the EPM with female gerbils showed similar results to those found in rats and mice – i.e., gerbils avoided the open arms and prior treatment with diazepam produced increased exploration of those open areas (Varty et al., 2002a). However, male gerbils exhibited high open-arms exploration, despite exposure to a long session of 20-min to the EPM (Rico et al., 2016), and explored the open arms more than rats did (Wang et al., 2018).

Despite the wide implementation of the EPM to assess anxiety-related behaviors in rodents, the interpretation of these measures in gerbils has received limited attention. For instance, differences between gerbils and other rodents in species-specific defense reactions related to exploration of open and closed areas have been not tested.

Gerbils are social rodents commonly found in desert habitats, which live in subterranean nesting burrows interconnected by tunnels (Ågren et al., 1989). Both aerial and ground threats elicit defensive responses of gerbils located on the surface (Ågren et al., 1989; Kotler et al., 1993). Ellard (1996) analyzed these defensive responses using an open field in which gerbils were exposed to six presentations of overhead visual stimuli that resembled an aerial predator. Whereas first presentation of the aerial threat triggered a fleeing response, repeated presentation of this stimulus attenuated this response. Ellard (1996) interpreted this effect as the gerbils’ habituation to the repeated association of a threatening stimulus with an increasingly familiar context. In addition, Ellard (1996) proposed that such attenuation of fleeing is the most adaptive response in a situation in which no shelter is available. When gerbils had access to a safe refuge (an attached enclosure in the open field with a solid roof), they spent extended periods inside of it.

The results of the light/dark test suggest that a dark area does not necessarily represent a safe refuge for gerbils. While rats of the vehicle group exposed to light/dark test spent almost the entire session in the dark compartment (98%; Chaouloff et al., 1997), gerbils spent less than half of the session in the dark area (Bridges and Starkey, 2004; Bradley et al., 2011). In addition, gerbils spent more time in the central area of an open field when compared with rats (Wang et al., 2018). These results suggest that in laboratory conditions, gerbils prefer protected areas that resemble the burrows in which they live in natural environments (enclosure with a solid roof) but not dark areas without roof, such as the closed arms of the EPM.

Research with other experimental preparations has also shown differences between gerbils and other rodents in terms of their defensive behavior. In fear conditioning and avoidance tests with shocks, rats freeze considerably more than gerbils, which results in better performance of rats in inhibitory tasks such as step-down and step-through avoidance. Conversely, gerbils show better adjustment to shuttle and lever-press avoidance, which entail active responses, including movement across the instrument (Ashe and McCain, 1972; Galvani et al., 1975; Powell et al., 1978; Crawford et al., 1981; Hurtado-Parrado et al., 2017).

Novelty and stress in the form of exposure to an strange and unprotected environment is one of the most effective means of triggering seizures in gerbils, whereas habituation reduce seizure frequency (Kaplan, 1975; Ludvig et al., 1991; Bertorelli et al., 1995). The fact that gerbils often exhibit spontaneous seizures in the EPM (Bridges and Starkey, 2004; Starkey et al., 2007; Rico et al., 2016) suggests that exposure to this instrument forces naïve gerbils to explore a novel environment that entails a conflicting situation in which closed arms do not provide a safe area, whereas open arms could offer an escape route.

Considering the documented defense reactions of intact gerbils to novelty and open/closed areas, the present study aimed to determine the effect of the benzodiazepine diazepam (DZP) on the spatiotemporal behavioral patterns of male gerbils exposed to an extended 20-min EPM session.

## Materials and Methods

### Subjects

Forty-two outbred male 10-weeks old Mongolian gerbils (50–70 g; *Meriones unguiculatus*), obtained from the National Institute of Health at Bogotá-Colombia, were used in this study. Animals were housed in groups of 2–3 in polycarbonate cages (42 × 20 × 20 cm) which contained dust free wood shaving bedding, and were kept in an animal room under a 12 h light/dark cycle (lights on at 08:00 h) with water and standard rodent pellets available *ad libitum*. The room temperature was maintained at 23°C with 55% relative humidity. Animal handling was limited to home cage-cleaning time and the animals were not habituated to a novel environment before the first test session. All experimental procedures were performed in accordance with the United States National Institute of Health Guide for the Care and Use of Laboratory Animals and were approved by our Institutional Animal Care and Use Committee (CICUAL-KL/COM43-2016).

### Apparatus

An EPM similar to that previously described elsewhere (Starkey and Bridges, 2010; Rico et al., 2016) was used in this study. The apparatus consisted of two open arms (40 cm × 8 cm) at right angles with two closed arms of the same size, with 30-cm high black Plexiglas walls. Arms emerged from a central platform (8 cm × 8 cm). The floor of the plus-maze was made of black smooth Plexiglas and the entire apparatus was elevated 50-cm from the ground. A raised Plexiglas edge (0.5-cm) surrounded the open arms to prevent gerbils from falling. The level of illumination of the test room was adjusted to 30 lux measured at the central area of the plus-maze.

### Drugs

The benzodiazepine DZP (0.5 mg/kg; Roche, São Paulo) was dissolved in physiological saline (NaCl 0.9%; Vehicle). Thirty minutes before placing the gerbil in the EPM, DZP or Vehicle was administrated intraperitoneally (i.p.) in a volume of 1 ml/kg. Drug doses and administration protocol were based on those used in similar studies using gerbils (Varty et al., 2002a; Bradley et al., 2007a,b, 2011).

### Behavioral and Scoring Procedures

In rats, the aversion to the open arms seems to be influenced by procedural factors, such as the time of the day at which testing occurs (Griebel et al., 1993). In gerbils, testing in the EPM has been performed between 8:00 and 16:00 h (Bridges and Starkey, 2004; Starkey and Bridges, 2010). Accordingly, our experimental sessions were carried out during the light phase (08:00–13:00 h). In order to characterize the spatiotemporal behavioral pattern during the session and to obtain behavioral dimensions that emerge from factor analysis, the sample of control group was expanded. Thirty minutes prior to testing, each animal was removed from the home cage, weighed and injected intraperitoneally with DZP (0.5 mg/kg; *n* = 9) or with Vehicle (*n* = 33). Then, gerbils were placed in the central area of the EPM facing one of the open arms, and could freely explore the instrument for 20-min. At the end of each session, the maze was cleaned with a 10% ethanol solution and dried with a cloth. All behavioral tests were recorded with a video camera placed above the EPM and connected to a digital video recorder in an adjacent room. A trained observer, blind for treatment, analyzed the videos (intra-observer agreement >90%). Behavioral measures were scored using the ethological free software X-PloRat (Tejada et al., 2017). The frequency of entries and the time spent into the open and the closed arms, the number of crossed squares into each arm and the frequency of stretching attend posture (SAP; a movement where gerbil leans forward with a flattened back followed by retraction to original position), were analyzed. An entry into an arm or into a square (8 cm × 8 cm) within an arm was scored after all four paws of the gerbil entered it. When an animal jump from the EPM, it was replaced on the central area as soon as possible facing one of the open arms, and additional time proportional to the time the subject was off the maze was added to the session. In these cases, video scoring was paused until the animal was repositioned in the EPM.

### Data Analysis

Gerbils occasionally exhibit seizures when exposed to the EPM, often followed by a period of immobility (Bridges and Starkey, 2004; Rico et al., 2016). Six animals from the control group that displayed seizures were excluded from the analyses.

In order to characterize the spatiotemporal behavioral pattern across an extended EPM session, data from 33 animals of the vehicle group were analyzed minute-by-minute and 5-min bins. Then, a factor analysis was performed for each of the 5-min bins to capture the behavioral dimensions that emerge across different segments analyzed with this session. Finally, the effects of DZP on the behavioral response of gerbils during the session were analyzed according to the factors that were identified. Behavioral parameters from vehicle group were expressed as mean ± SEM and submitted to Friedman repeated measures (RM) analysis of variance (ANOVA) on ranks. The factor analysis was performed by principal-component followed by an orthogonal Varimax rotation. Factors with eigenvalues greater than 1.0 and loadings greater than 0.7 were kept. The measures analyzed with this procedure were the percentage of time spent in the open arms and in the central area; the percentage of entries in the open-arms and the closed-arms entries; the distance traveled in the closed-arms and the frequency of SAP in the central area. Data from the effects of DZP were expressed as mean ± SEM and submitted to Friedman RM-ANOVA on ranks for intra-group analysis. For each minute or for each 5-min bin, comparisons between the DZP and vehicle group were made by means of Mann-Whitney rank sum test. Whenever necessary, the SNK *post hoc* test were used. In all cases, the significance level was set at *p* < 0.05.

## Results

### Vehicle Group

**FIGURE 1:**
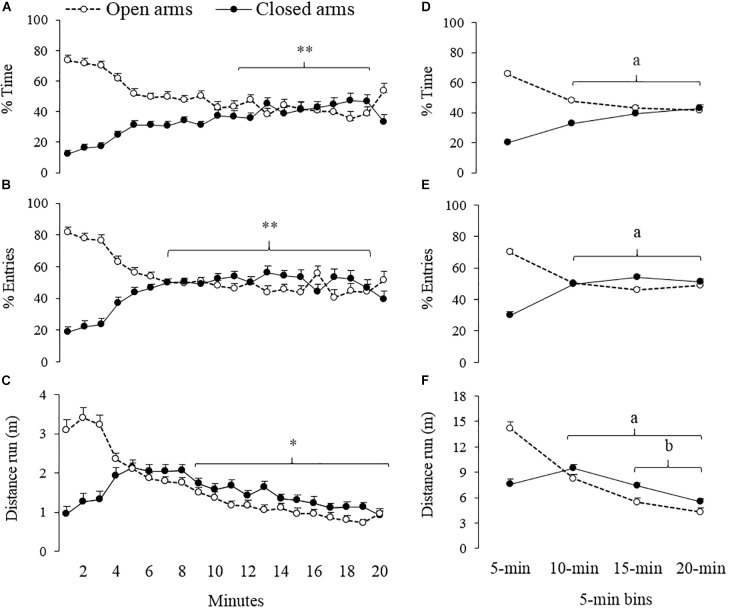
Spatio-temporal analyses of exploratory behavior of gerbils (*n* = 33) exposed to 20-min session in the EPM. Left panels **(A–C)** show minute-by-minute session data and right panels **(D–F)** the same data in 5-min bins. ^∗∗^different from 1 to 3-min for open and closed arms, ^∗^different from 1 to 5-min for open arms, ^a^different from 0 to 5-min for open and closed arms, ^b^different from 6 to 10-min for open and closed arms (*p* < 0.05).

Results of temporal analysis on vehicle group data are summarized in Figure 1 and Table 1. Eleven animals jumped from the open arms of the EPM during the first minute of exposure to the apparatus. As described in method section, subjects were replaced in the central area and session time was adjusted. For the percentage of time spent and percentage of entries in the open and closed arms, RM-ANOVA on ranks showed differences between the intervals in which the session was divided (min-by-min and 5-min bins; Table 1).

**Table 1:** Summary of statistical parameters from minute by minute and 5-min bins analysis of the session time in the EPM.

Behaviors	Min-by-min		5-min bins	
	*χ2*_(19)_	*p*	*χ2*_(3)_	*p*
**Vehicle group (Figure 1)**				
% Time (s)				
Open arms	124.45	< 0.001	43.36	< 0.001
Closed arms	113.97	< 0.001	53.76	< 0.001
% Entries				
Open arms	132.54	< 0.001	43.46	< 0.001
Closed arms	121.22	< 0.001	43.46	< 0.001
Distance run (m)				
Open arms	315.91	< 0.001	73.11	< 0.001
Closed arms	104.05	< 0.001	75.56	< 0.001
**Vehicle vs. diazepam (Figure 2–4)**				
% Open-arms time (s)				
Vehicle	124.45	< 0.001	43.36	< 0.001
Diazepam	77.97	< 0.001	16.60	< 0.001
% Open-arms entries				
Vehicle	132.54	< 0.001	43.46	< 0.001
Diazepam	86.43	< 0.001	17.13	< 0.001
Closed-arms entries				
Vehicle	177.47	< 0.001	48.06	< 0.001
Diazepam	95.24	< 0.001	16.06	0.001
Closed-arms distance run				
Vehicle	104.05	< 0.001	37.66	< 0.001
Diazepam	63.42	< 0.001	11.40	0.001
Central area SAP				
Vehicle	30.49	0.046	12.453	0.006
Diazepam	36.76	0.008	14.733	0.002
% Central area time (s)				
Vehicle	64.65	< 0.001	22.16	< 0.001
Diazepam	75.99	< 0.001	17.13	< 0.001


*Post hoc* minute-by-minute tests indicate that animals spent more time in the open arms during the first 3 min of the session, as compared to the segment between the 11th and 19th minute (Figure 1A). Similarly, the number of entries to the open arms was higher during the first 3 min than during minute 7 through 19 (Figure 1B). *Post hoc* minute-by-minute tests also showed an opposite pattern compared to the closed arms; namely, less time spent and fewer numbers of entries during the first 3 min of the session as compared to later segments. A further analysis based on 5-min segments showed significant differences between the first bin and the rest of the segments for both measures (time spent and entries) and both types of arms (open and closed) – See Figure 1D,E. RM-ANOVA on ranks also showed differences in the distance that the gerbils ran in the EPM across different moments of the session (Table 1). *Post hoc* minute-by-minute tests showed that the gerbils reduced the locomotion in the open arms during the last 11 min of the session as compared to the segment between the 1th and 5th minute (Figure 1C). *Post hoc* tests also indicated that across the de 5-min bins, gerbils’ locomotion gradually decreased (Figure 1F).

### Factor Analysis

Factor analysis results are summarized in Table 2. For each of the four 5-min bins of the session, a factor analysis was performed. Behavioral parameters related to open and closed arms exploration, as well as time spent and stretching attended posture (SAP) in the central area of the maze were processed using principal-component analysis. After an orthogonal Varimax rotation, a single factor representing the 80.9% of the variance emerged for the first 5-min of the session. For that single factor, open-arms exploration measures were negatively correlated with closed-arms and central area exploration. For the following three bins, three factors emerged explaining 88.3, 86.1, and 85% of the variance, respectively. A similar pattern of factor solution was observed for the last three 5-min bins of the session. One factor grouped the open-arms exploration measures, while the other two factors grouped measures related to closed-arms exploration and central area activity (Table 2).

**Table 2:** Orthogonal factor loadings obtained from control group gerbils for the first 5-min and the following 5-min of the session in the elevated plus-maze test.

Behaviors	First 5 min	6 to 10 min		
	Factor 1 Escape-related behaviors	Factor 1 Anxiety-related behaviors	Factor 2 Approach to the closed arms	Factor 3 Risk-assessment behaviors
% Open arms time (s)	–0.97	0.79		
% Open arms entries	–0.93	0.91		
Closed arms entries	0.94		0.87	
Closed arms distance run (m)	0.94		0.91	
% Central area time (s)	0.82			0.87
Central area SAP	0.79			0.90
% of variance	80.90	27.06	27.99	33.28


*Principal-component analysis was followed by an orthogonal Varimax rotation. Factors with eigenvalues greater than 1.0 and loadings greater than 0.7 were kept. Minus signs (-) indicate the direction of the particular loading. A single factor representing the 80.9% of the variance emerged for the first 5-min of the session. For that single factor, open-arms exploration measures were negatively correlated with closed-arms and central area exploration. After a 5-min period (6–10 min), the EPM test measured three independent defensive behaviors, namely anxiety (% of time and entries to the open arms), approach to the closed arms (entries and distance traveled), and risk assessment (% of time in the central area and SAP).*

### Effect of DZP

**FIGURE 2:**
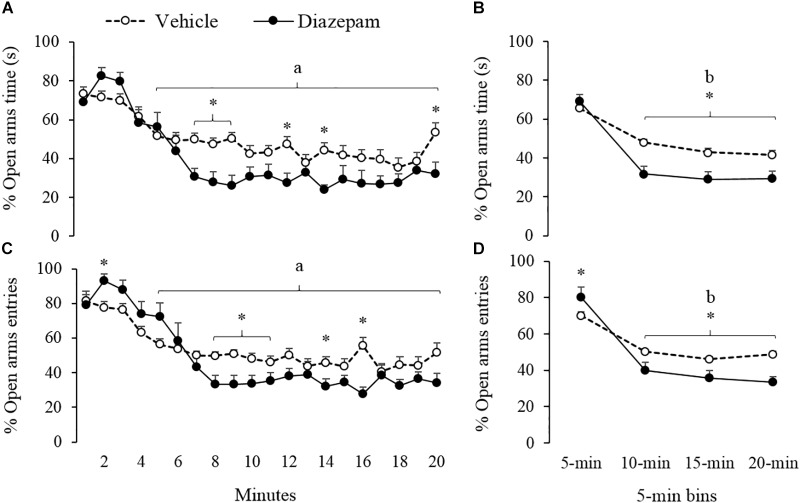
Effect of DZP on escape-related behaviors of gerbils exposed to 20-min session in the EPM – i.e., percentage of time and percentage of entries. The left panels showed minute-by-minute data (Panel **A**, **C**) and right panels show data in 5-min bins (Panel **B**, **D**). After 5-min of exposure to the EPM, DZP reduced escape-related behaviors. ^a^different from minute 1 to 4 for vehicle and DZP group, ^b^different from 5-min bin for vehicle and DZP group, ^∗^different from vehicle group (*p* < 0.05).

**FIGURE 3:**
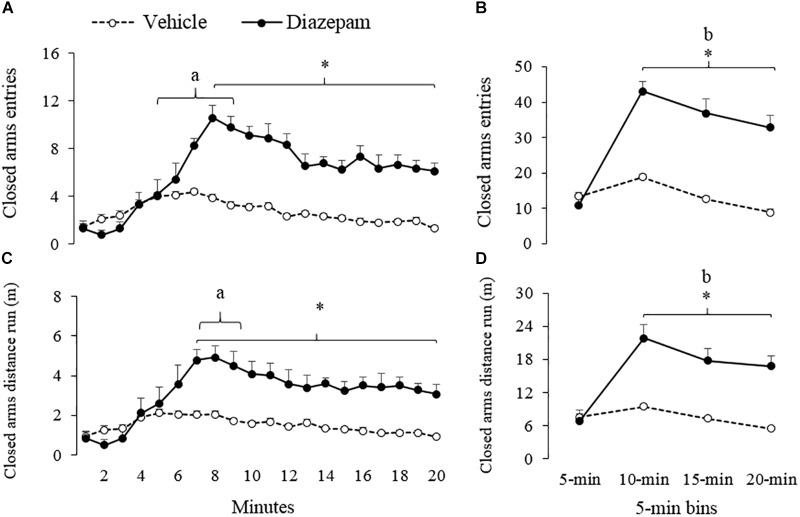
Effect of DZP on the approach to the closed arms of gerbils exposed to 20-min session in the EPM – i.e., percentage of entries and distance run (m). The left panels show minute-by-minute data (Panel **A**, **C**) and right panels show data in 5-min bins (Panel **B**, **D**). After 5-min of exposure to the EPM, DZP increased the approach to the closed arms of the maze. ^a^different from minute 1 to 4 for vehicle and DZP group, ^b^different from 5-min bin for DZP group, ^∗^different from vehicle group (*p* < 0.05).

**FIGURE 4:**
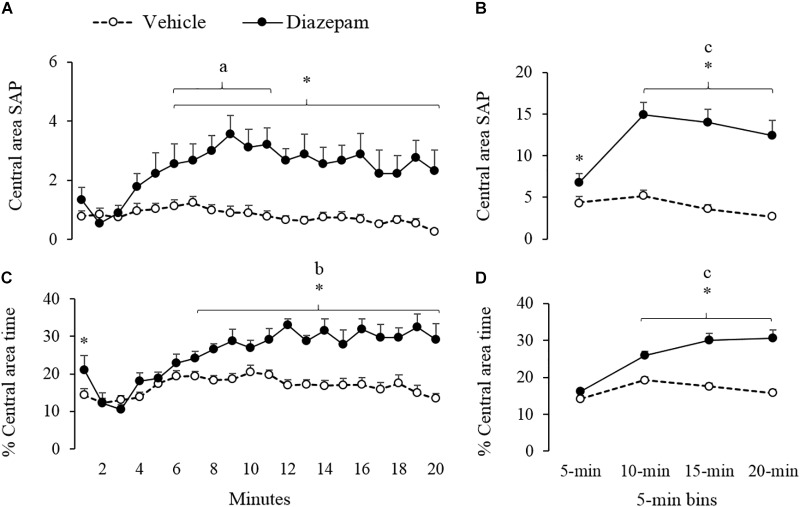
Effect of DZP on risk-assessment behaviors of gerbils exposed to 20-min session in the EPM – i.e., SAP and percentage of time. The left panels show minute-by-minute data (Panel **A**, **C**) and right panels show data in 5-min bins (Panel **B**, **D**). After 5-min of exposure to the EPM, DZP increased the risk-assessment behaviors. ^a^different from the 2nd minute for DZP group, ^b^different from the 2 to 5 min for DZP group, ^c^different from 5-min bin for DZP group, ^∗^different from vehicle group (*p* < 0.05).

The effects of the anxiolytic compound DZP on the behavioral response of gerbils are summarized in Figure 2–4 and Table 1 (Vehicle vs. DZP). RM-ANOVA indicated differences between the bins into which the session was divided for the open arms (percentage of time and entries), the closed-arms (entries and distance run) and the central-area activity-related measures (percentage time and SAP; Table 1). *Post hoc* tests of minute-by-minute data showed that starting in the fourth minute of the session, gerbils treated with vehicle and DZP significantly reduced open-arms exploration in terms of percentage of time and entries (Figure 2A,C, respectively).

Similarly, a 5-min bin analysis showed significantly lower time allocation and entries to the open-arm during the last three bins as compared to the first bin (see Figure 2B,D). *Post hoc* tests also showed that compared to the first 5-min, gerbils treated with DZP increased both closed-arms exploration and central-area activity during the last 15 min of the session (Figure 3, 4). A Mann-Whitney test was used to compare vehicle and DZP groups. It showed that during the second minute and the first 5-min bin, DZP-treated gerbils increased significantly the percentage of entries to the open arms (*p* < 0.05) compared to vehicle group (Figure 2C,D). In addition, during the first 5-min bin DZP also increased SAP frequency (*p* < 0.05). A Mann-Whitney test for the last 15-min of the session, indicated that DZP-treated gerbils reduced significantly (*p* < 0.05) time allocation and entries to the open-arms, as compared to vehicle group (Figure 2). Lastly, DZP significantly increased (*p* < 0.05) gerbils’ entries and distance run in the closed arms, and time spent and SAP frequency in the central area (Figure 3, 4).

## Discussion

In the present study, we analyzed the effect of the anxiolytic DZP on the defensive behavior of gerbils exposed to a 20-min EPM session. In addition, data of the vehicle group (*n* = 33) were analyzed minute-by-minute and in 5-min bins to characterize the spatiotemporal behavioral pattern across the session. Lastly, a factor analysis was conducted to determine the behavioral dimensions related to the EPM test.

Gerbils in the vehicle group showed a different pattern of defensive responses during two stages of the session: avoidance of the closed arms and preference for the open arms during the first 5-min, followed by homogeneous exploration of open and closed areas during the remaining of the session. Animals treated with DZP showed increased locomotion, risk assessment, and closed-arms exploration.

This is the first temporal analysis of the effects of DZP on the defensive behavior of gerbils exposed to an extended 20-min EPM session. The results of the first 5-min of the session are not consistent with observations in other rodent species – i.e., rats and mice exposed to a standard 5-min EPM session avoided the open arms, and anxiolytic treatment lead to increased exploration of those areas (for a review see Carobrez and Bertoglio, 2005; Pawlak et al., 2012). To interpret our results, species-specific differences in defensive reactions between gerbils and other rodents were considered. We propose that exposure to the EPM activates the gerbil’s defensive system, in preparation for aversive events such as the potential attack of an aerial or ground predator. It appears that gerbils alternate defensive responses when confronting a new environment, such as the EPM. During the first 5 min of the session, gerbils primarily show escape behavior throughout the open arms; in fact, despite the use of a raised edge surrounding the open arms to prevent gerbils from falling, one third of the control animals escaped from the EPM jumping from these areas. Similar to gerbils, Holmes et al. (2000) reported that three of fourteen male wild mice also jumped from the plus-maze when placed on the apparatus. When confronting imminent predation in a new environment, that entails open and partially protected areas (uncovered closed arms), it seems that the most adaptive defensive response that gerbils could display is identifying and pursuing escape routes from the instrument – i.e., fleeing via the open arms. Conversely, closed arms do not seem to be a good option as they do not represent safe refuge and actually obstruct escape due to the walls.

This approach could explain the behavioral patterning of the gerbils during the first 5 min of the session – i.e., avoidance of the closed arms and preference for the open arms and it is consistent with observations under other controlled situations. For instance, during encounters with snakes in a maze, gerbils showed overall more exploratory behavior than rats, and identified the exits and safe or dangerous places of the apparatus within the first minutes of the session (Guimarães-Costa et al., 2007). Moreover, when confronted simultaneously with aerial and ground threats, gerbils reduced the use of protected areas in favor of the open sections of an outdoor aviary (Kotler et al., 1992).

Though this gerbils’ tendency to avoid the closed arms and prefer the open areas of the EPM is consistent with the results obtained in other studies (Bradley et al., 2007a,b; Starkey and Bridges, 2010; Rico et al., 2016), Varty et al. (2002a,b) found that female gerbils of a control group avoided open-arms and preferred closed arms. A possible explanation for this discrepancy may be related to the characteristics of the instrument that was utilized. Whereas in Varty et al’s study the walls of the closed arms were clear to allow for constant illumination in all parts of the maze (Varty et al., 2002a,b), we used an apparatus similar to those described elsewhere in which the closed arms were surrounded by black walls (Bradley et al., 2007a,b; Starkey and Bridges, 2010; Rico et al., 2016). It seems that the use of dark instead of clear walls in the closed arms affect the defensive response of gerbils in the EPM.

Having elapsed 5 min of the session, gerbils reduced exploration and the escape response via the open arms. It is possible that during this period of habituation animals learn that, due to the height of the open arms (50 cm), the escape response is not functional. Accordingly, gerbils start to use the closed arms for refuge, without fully abandoning the escape attempts through the open arms. This interpretation would explain the homogeneous pattern of exploration of open and closed arms observed after the fifth minute of the session, which is consistent with the habituation of the escape response reported by Ellard (1996).

### Factor Analysis and Effect of DZP During the First 5 min of the Session

This is the first study to report a factor analysis of a 5-min EPM session for gerbils. Whereas behavioral measures in rats and mice (Wall and Messier, 2001) tend to group into at least three independent factors related to anxiety (exploration of open arms), locomotion (exploration of closed arms), and decision making/risk assessment (time in the central area and SAP), in our study a single factor emerged. The factor solution observed in gerbils suggests that during the first 5 min of the session, the EPM test is measuring a single defensive behavior characterized by a negative relationship between exploration of open arms (percentage and number of entries) and closed-arms (number of entries and distance traveled), and time in the central area and SAP. Similar to the escape response described by Ellard (1996), during the first 5 min of the session gerbils showed vigorous locomotion in the open arms, which included bursts of high-speed running in the absence of risk-assessment and visits to the closed arms. Accordingly, it seems that during the standard 5-min session the measures obtained in the EPM are mostly related to the escape behavior and not to anxiety-related responses.

Animals in the vehicle group exhibit a high percentage of entries to the open arms (69.9%), which was increased by the DZP treatment (80.3%). Our findings suggest that DZP facilitates the escape response through the open arms during the first 5 min of the session. Though Bradley et al. (2007a) also reported an increase in the percentage of entries to the open arms in DZP-treated gerbils, Varty et al. (2002a) reported that the same anxiolytic increased the time spent but not the number of entries to the open arms. Again, these inconsistencies with Varty et al’s results may be related to the characteristics of the EPM that was utilized (clear closed arms) and the sex of the gerbils (females).

### Factor Analysis and Effect of DZP After the First 5 min of the Session

Factor analysis of the subsequent 5-min bins of the session revealed three independent factors that grouped measures of open- and closed-arms exploration, and activity in the central area, respectively. These findings suggest that after a 5-min exploration period, the EPM test measures three different behavioral dimensions. According to our interpretation, once habituated to the instrument, gerbils may face a conflict between finding shelter in the closed arms – notwithstanding these areas do not represent an entirely safe refuge – or continue trying to escape via the open arms. We propose that the identified factors possibly correspond to three independent defensive behaviors, namely anxiety (% of time and entries to the open arms), approach to the closed arms (entries and distance traveled), and risk assessment (% of time in the central area and SAP). During the last three 5-min bins of the session, gerbils treated with DZP reduced their time spent and entries to the open arms, while increased their exploration of closed-arms, the time in the central area, and incidence of SAP. These effects of DZP on the defensive behavior of gerbils are different to those reported for mice and rats (for a review see Carobrez and Bertoglio, 2005).

It has been proposed that the EPM is a conflict test in which a novel situation produces both approach (open-arms) and unconditioned avoidance (closed arms), interpreted as curiosity and caution, respectively (Handley and McBlane, 1993; Ohl, 2005). In this context, anxiolytic compounds such as DZP can shift the balance of this conflict from avoidance toward approach (Gray and McNaughton, 2000/2003). Although further tests of our interpretations are needed, we propose that the conflict produced by the presentations of open and closed areas appears later in the session, once gerbils have habituated to the EPM. This conflict would consist on the tendency to continuous attempts to escape via the open arms and finding shelter in the closed arms. Accordingly, DZP would rapidly reduce the escape response through the open arms, while increasing approximations to the closed arms and risk-assessment that precedes the visits to the open arms.

Our results also indicate that after 5 min in the EPM, DZP-treated gerbils increase their locomotion in the closed arms. Apparently, this effect of DZP is related to the gerbils’ defensive response to novelty; it has been shown that gerbils’ reactivity to novel environments interferes with the effect of diverse compounds, included DZP (Babcock et al., 2002; Okano et al., 2005; Prinssen et al., 2006). Moreover, gerbils treated with DZP increase locomotion in new environments, but not in familiar settings (Prinssen et al., 2006).

Our results indicate that male *naïve* gerbils require sessions of at least 10 min in the EPM in order to properly measure anxiety-related behavior in this model. Data from this session should be analyzed in two 5-min bins; during the first 5 min (standard-session length), the obtained measures seem to be related to a vigorous escape response via the open arms triggered by the novel environment. Once habituated to the instrument, the escape response decreases, and approximations to the closed arms increase. Gerbils seem to face a conflicting situation between continue attempting to escape via the open arms or find shelter in the closed arms, notwithstanding these areas do not offer entirely safe refuge as they are not covered. This conflict appears to be reduced by DZP treatment, which produces a reduction in the escape behavior and increases both approximations to the closed arms and risk assessment. Accordingly, anxiety-related measures obtained in the EPM for gerbils could be only observed after the 5-min habituation period. Unlike in rats and mice, decrements in exploration of open-arms and increments in risk-assessment could be interpreted in gerbils as an anxiolytic effect. Further studies are necessary to test the role of procedural factors such as the effect of DZP in EPM-experienced gerbils, with or without prior habituation to the experimental room or the experimenter (manipulation).

## Data Availability

All datasets generated for this study are included in the manuscript and/or the supplementary files.

## Author Contributions

JR and ML developed the study concept and design. LM-T performed the data collection. JR and LM-T performed the data analysis. JR, ML, and CH-P contributed to the interpretation of the data and provided important critical revisions. CH-P contributed to style of language in English writing. All the authors approved the final version of the manuscript.

## Conflict of Interest Statement

The authors declare that the research was conducted in the absence of any commercial or financial relationships that could be construed as a potential conflict of interest.
